# Perioperatory pain in oncological patient - physiopathological and therapeutical implications

**Published:** 2014

**Authors:** C Diaconu, C Pantis, C Cirimbei, C Bordea, A Blidaru

**Affiliations:** * Anaesthesia and Intensive Care Unit, Oncological Institute Bucharest; ** Surgery Clinic No. 1, Oncological Institute Bucharest; *** Surgery Clinic No. 2, Oncological Institute Bucharest

**Keywords:** perioperatory pain, multimodal treatment, inflammatory mediators, hormones

## Abstract

Perioperatory pain in oncological patients represents a witness of anesthetic-surgical aggression, frequently exacerbated by the complementary radio-chemotherapy and also a predictive factor for postoperatory evolution. The objectivation of perioperative pain by scales of clinical evaluation does not offer a certain and objective quantification; so, the dosing of some hormonal and acute phase inflammation mediators could realize a more realistic projection. Clinical and biological correlation can offer a support for an adequate and well-balanced treatment.

Divinum est sedare dolorum

Blessed are those who treat pain.

Galen

According to the last studies, pain represents an important predictive factor of the patients’ postoperatory evolution, therefore its control represents an important protective factor for postoperatory healing [**1**,**2**]. It is unanimously accepted that in the absence of an adequate pain control, an ill-fated cascade of events such as cardiopulmonary events (tachycardia, hypoxia), digestive events (nausea, vomiting), psychic events (anxiety, depression) are triggered, carried and amplified, as well as the healing capacity and immunity impairment (including the antineoplastic one). All these events are included in the surgical postaggressional stress reaction, which expresses itself through sympathetic nervous system activity increasing, catabolic hormones excessive secretion and hypercoagulability.

The oncological patient, exposed to perioperatory anesthetic-surgical stress, represents a special category from the point of view of algetic syndrome presence. This exposure is also on short and long term, of over 6 months (chronic pain), syndrome justified by the disease, the surgical procedure, as well as the adjuvant and neo-adjuvant radiochemotherapy therapies. The syndrome known as Persistent Post-Surgical Pain (PPSP) can develop in oncological patients who have undergone surgery, especially if the acute pain control is defective [**3**].

In its untreated acute form, the persistence of pain during the postoperatory period can trigger a myocardial ischemia until infarction, especially when it appears in anemic patients with preexistent coronary impairment, as a consequence of tachycardia, elevated blood pressure and elevated cardiac activity. The decrease of blood flow in periphery, because of vascular resistance increase, impairs the healing capacity of the postsurgical parietal scar. When pain is felt in the thoracic region, a respiratory function limitation appears, with a tendency to atelectasis, followed by pulmonary infections and hypoxemia. The patients’ reflex of antalgic movements limitation expose them to prolonged immobility and an important increase of thromboembolic risk. The central hypothalamic-pituitary chain with catabolic hormones secretion, with protein destruction and glycaemic elevation is also activated, which also affects the postoperatory healing capacity.

Perioperatory pain triggered by painful and inflammatory stimuli, brutally installed during the surgical procedure but constantly present in plateau in oncological patients, determines mediators unloading with pain mechanisms activation on central path (cytokines, interleukin-1-beta) and peripheral path (arachidonic acid activation cascade **Fig. 1**), both contributing in various percentages to the support of pain and its components, acute and chronic [**4**]. Because of this reason, the principle and the necessity of the multimodal pain treatment, with concomitant administration of active substances on both pain-triggered paths, emerged. Among the main pain mediators in the oncological patients exposed to anesthetic-surgical aggression, there are the tumor necrosis factor (TNF), somatostatin, cholecystokinin, serotonin, histamine, prostaglandins, calcitonin, GABA, mediators freed by cellular destruction, by eliciting capillary permeability by metabolic and local enzymatic disturbances.

**Fig. 1:**
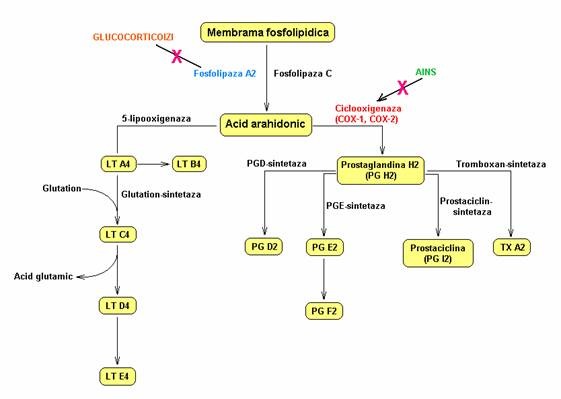
Arachidonic acid cascade

Pain perception has a different intensity for every subject, especially for acute pain. There is a “pain threshold”, resulted from the intrication of multiple subjective factors that reunite the anterior painful experiences, instruction and education degree, behavioral profile (anxiety elements, depression, and phobias). Pain intensity objectivation is performed by several clinical evaluation scales, three of them being mostly used: VAS (Visual Analogical Scale), VDS (Visual Descriptor Scale) and VNRS (Verbal Numerical Rating Scale), but all are marked by subjectivity and the patient’s understanding capacity. The subjects usually nominate 0 value of the scale, expressed numerically (VNRS) or in millimeters (VAS), respectively painless status and the maximal value 10 (VNRS) or 100 (VAS), corresponding to the maximal unbearable pain. The easiest scale to use is VDS, which conventionally stratifies pain in 3-4 stages: absent, moderate, medium or unbearable. The use of this pain quantification forms in oncological operated patients, in the ICU, is difficult and does not constitute a real, objective support for the accomplishment of analgesic therapeutics schemes. The necessity of pain objectivation is important for the establishment of analgesic types and doses, for some of them, besides the beneficial, protective effect, the secondary effects also exist, which negatively interfere with the postsurgical patient evolution, on digestive, pulmonary, respiratory, renal line, even on immunological, anti-infective and antineoplastic lines.

Regarding this issue, the fact that prolonged administration of opioid drugs (morphine, fentanyl) has been unanimously accepted to determine a decrease of the cell- and antibody-mediated immunity, as a consequence of the activity alteration of T-Natural Killer lymphocytes, of cytokines and phagocytosis, as it emerged from a very consistent review realized by Ramsin Benyamin in 2008 [**5**]. The same substances induce a marked depression of respiratory function, with a risk of ventilatory support necessity in the postsurgical period, as well as a digestive motility inhibition, with delayed intestinal transit recovery and digestive tolerance.

The modern pain therapy schemes during the postoperatory period, reunited under the concept of “Multimodal Analgesia”, aim for the quantity and type of administered drugs, as well as for the administration of paths, reported to surgical procedure type, comorbidities, patient weight, etc. Combinations of paracetamol, non-steroidal anti-inflammatories, anticonvulsants and antidepressants associated with major analgesics like morphine, oral opioids (tramadol, oxycodone, codeine, etc.) or fentanyl are performed, which can be administered on different ways: parenterally, per os, intravenously, intramuscularly or subcutaneously or even transdermally; the possibilities of loco-regional analgesia are not omitted either [**6**]. The principle of these analgetic combinations is to act synergically on the two pain-producing paths, central and peripheral, simultaneously combating different triggered pain stimuli and to intensify the effect of different analgesics, with dose diminishing especially for opioids.

All these grounds lead to the necessity of establishing a real equilibrium between the analgesic and pain, naturally with its complete absence, but with optimal and adequate doses of analgesics, in order to combat the algetic postoperatory stress, but also to prevent the negative effects of excess administration of analgesics. The objectivation of the pain level and its implicit control, can be performed by the determination of a biological markers complex including inflammation factors (C-reactive protein, ESR, thrombocytes, interleukins - 1 and 6, ceruloplasmin), hormonal factors (cortisol, thyroid hormones, ADH, etc.).

The anesthetic-surgical aggression and postoperatory consecutive status trigger as a part of stress body reaction, multiple hormonal changes, released especially on the hypothalamic-pituitary and sympathetic-adrenergic axis, having as main effects the hypercatabolism, with the generation of energetic substrate, conservation of water and electrolytes capital, with the purpose of maintaining the cardiovascular parameters [**7**].

Significant increases of cortisol values (up to 3-4 times over the basic plasmatic level), small increases of glucagon correlated with a moderate decrease of insulin, and unexpectedly a small decrease of thyroid-hormones level (possibly determined by hypercortisolemia) appeared. Additionally, quantifiable increased values also expressed the antidiuretic hormone (ADH), neurotensin and beta-endorphin levels as well as the growth hormone, aldosterone and ACTH. Cortisol, ADH and ACTH, with predictable pre- and postoperatory variations are frequently used as markers in the evaluation and control of pain.

There is a very well studied panoply regarding the pain correlation with the biological markers and mediators of inflammation, especially with factors implied in the acute phase of inflammation. Of the cytokines class, proteins synthesized by leucocytes, fibroblasts and endothelial cells, the interleukin IL-1 and interleukin IL-6, besides the TNF-alpha factor, represent markers of tissue lesions and implicit postoperatory pain [**8**]. Interleukin 6 also has important roles in the cellular and humoral immune response modulation (being a growth and differentiating factor of B-cells with immunoglobulin synthesis stimulation, as well as the activation, growing and differentiating of T-lymphocytes). Consequently, detectable plasmatic increases appeared as a reaction to bacterial and viral infections, inflammations and traumas, at levels over 3,8 pg/ml. At the same time, Interleukin-6 represents an induction element of hepatic synthesis of proinflammatory reactants of acute phase: fibrinogen, haptoglobin, C-reactive protein, complement. On the contrary, the same increase of IL-6 serum concentration generates a decrease of the hepatic synthesis of albumin and transferrin, with priming of hepatocitary regeneration. Well-based studies proved that IL-6 levels are lower after laparoscopic surgical procedures (cholecystectomies, hysterectomies), in comparison with open procedures, the mini-invasive approach being less traumatic and implicit with diminished post-operatory pain [**9**-**11**].

Also, the C-reactive protein and fibrinogen, that could also be benchmarks in the postoperatory pain evaluation are acute phase mediators in the category. Which is also interesting, is the evolution of some hepatic synthesized carrier proteins, whose synthesis is influenced in the acute phase of inflammation: albumin, transferrin and ceruloplasmin. The plasmatic levels of the first two have the tendency to decrease because of an anesthetic-surgical aggression and implicit decrease of plasmatic iron and zinc; on the contrary, the plasmatic level of ceruloplasmin increases, simultaneously with blood copper level. Ceruloplasmin, an alpha2-globulin, with a priority role in the transport of approximately 70% of the serum copper, is implied in antioxidative processes placed at the cell membrane level, but also with anti-inflammatory properties by serum histaminase inhibition. Due to this last feature, ceruloplasmin is a veritable acute phase reactant, increasing the plasmatic concentration accompanying the acute and chronic inflammatory processes, with values of over 60 mg/dl.

**Fig. 2:**
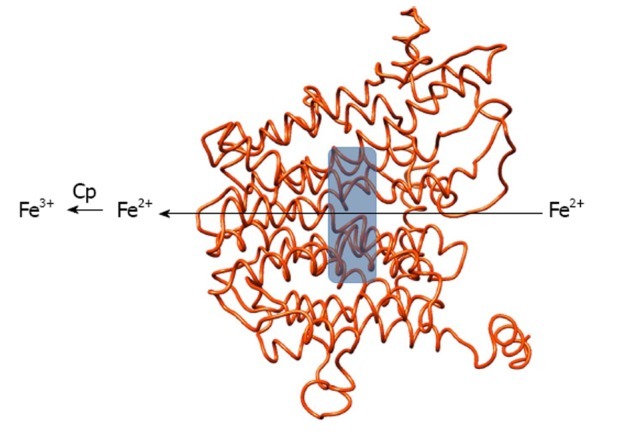
Structural model of human ceruloplasmin viewed along the membrane plane

The attempt of integrating the data harvested by clinical pain evaluation scales, with values of several serological parameters represented by hormones implied in stress and pain reactions, along with the biological markers of inflammation, can constitute the construction of some mixed evaluation scores of postoperatory pain evaluation, with the biological objectivation of painful reactions and consequently establishing well-balanced analgesic treatment schemes. This way, the use of excessive doses of major analgesics, burdened with a quite large variety of negative secondary effects, some of them less obvious are avoided. On the other side, in some patients categories, additional arguments for the intensification of therapy against pain can be found.
